# Inflammatory Choroidal Neovascular Membranes in Patients With Noninfectious Uveitis: The Place of Intravitreal Anti-VEGF Therapy

**Published:** 2020-03-25

**Authors:** Omer Karti, Sefik Can Ipek, Yesim Ates, Ali Osman Saatci

**Affiliations:** 1 Department of Ophthalmology, İzmir Democracy University, İzmir, Turkey.; 2 Department of Ophthalmology, Ağrı State Hospital, Ağrı, Turkey.; 3 Private Retina Specialist, İzmir, Turkey.; 4 Department of Ophthalmology, Dokuz Eylul University Medical Faculty, İzmir, Turkey.

**Keywords:** Aflibercept, Bevacizumab, Inflammatory Choroidal Neovascularization, Intravitreal Injection, Ranibizumab, Vascular Endothelial Growth Factor Inhibitors

## Abstract

Inflammatory choroidal neovascularization (iCNV) is an infrequent but an important cause of visual morbidity in patients with non-infectious uveitis and mostly occurs in intermediate or posterior uveitis. Punctate inner choroiditis, Vogt-Koyanagi-Harada disease and multifocal choroiditis are among the leading causes of uveitis entities resulting in iCNVs. The diagnosis and management of iCNVs still remain a challenge. Use of multimodal imaging techniques such as fluorescein angiography, indocyanine green angiography, optical coherence tomography (OCT) and OCT-angiography may be necessary for the diagnosis of iCNVs. The treatment algorithm is not straightforward for iCNV. While control of the active inflammation with steroids and/or immunosuppressive agents is a key to success, various adjunctive treatment modalities such as thermal laser photocoagulation, photodynamic therapy and surgical membrane removal were also co-administered previously. Nowadays, vascular endothelial growth factor (VEGF) inhibitors have become the most commonly administered adjunctive treatment option as they provide better anatomical and functional outcome and the recurrence rate of CNV is relatively low. We hereby reviewed important clinical studies and case series on anti-VEGF administration in iCNVs and briefly overviewed their results.

## INTRODUCTION

**Figure 1:**
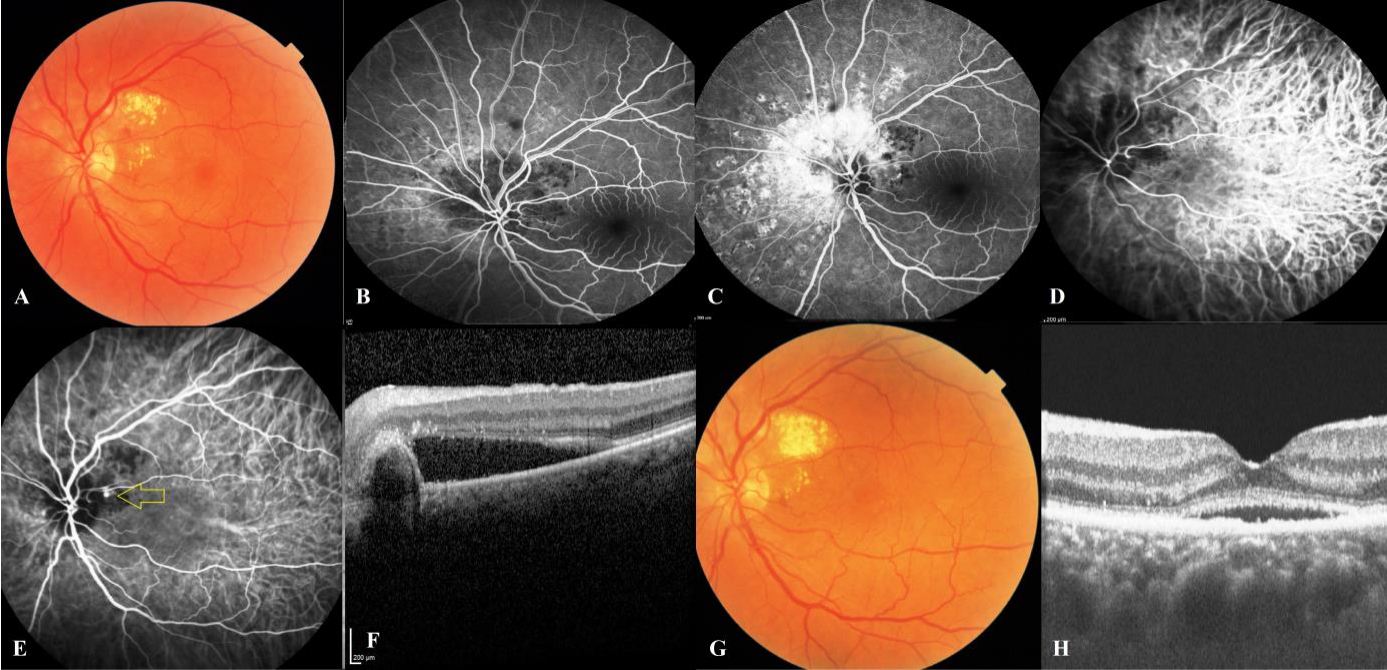
Images of a 64-year-old man with peripapillary healed multifocal choroiditis (MFC) scars and inflammatory choroidal neovascularization (iCNV). Right eye, color fundus picture showing peripapillary hard exudates and dot hemorrhages (A). Early venous phase of fluorescein angiogram showing a hypofluorescent lesion complex at the peripapillary area and multiple subtle hypofluorescent scattered dots (B) Late venous phase of fluorescein angiogram with profuse peripapillary hyperfluorescence (C), Early indocyanine green angiogram revealing peripapillary hypocyanesence of the lesion complex (D) and later frames showing peripapillary hypercyanesent lesion with a faintly seen dark halo suggestive of a ''polyp'' (yellow arrow) together with peripapillary hypocyanescent dots representing the old scars (E). Optical coherence tomography (OCT) showing subretinal fluid with sharp pigment epithelial detachment corresponding to the polyp (F). One month following thermal laser photocoagulation and anti-VEGF treatment, color fundus picture (G) and OCT (H) show some clinical improvement

Uveitis-related choroidal neovascularization (CNV) also termed as ''inflammatory CNV'' (iCNV) is a common cause of visual morbidity in uveitis, and the third leading cause of CNV following the exudative senile macular degeneration (SMD) and pathological myopia [1-3]. iCNV may develop during the course of both infectious and non-infectious types of uveitis [4, 5] and its incidence has been reported to be 2% in non-infectious posterior uveitis (NIPU) entities [5]. The incidence is especially relatively higher in some uveitis entities that affect or disrupt the Bruch’s membrane or neighboring layers. The highest risk of iCNV development in affected eyes has been reported in punctate inner choroiditis (PIC), Vogt-Koyanagi-Harada (VKH) disease and multifocal choroiditis (MFC) [5]. Its prevalence is reported to be 32-46% for MFC [6-9], 17-40% for PIC [4, 9, 10], 10-25% for serpiginous choroiditis (SC) [6, 11-16] and 9-15% for VKH disease [6, 17-19]. Besides common causes of iCNV aforementioned, CNVs can also be seen rarely in intermediate uveitis [20] or other NIPU entities including acute posterior multifocal placoid pigment epitheliopathy (APMPPE) [21], sympathetic ophthalmia (SO) [22], sarcoidosis [23], multiple evanescent white dots syndrome (MEWDS) [24] and birdshot retinochoroidopathy (BSCR) [25]. Though its exact pathogenesis remains unclear, the imbalance between stimulatory and inhibitory soluble mediators produced by the retinal pigment epithelium (RPE) seem to be the main triggering factor for its development [2]. Cytokines together with the vascular endothelial growth factor lead to impaired permeability and altered angiogenesis. Chronic inflammation induced Bruch's membrane-RPE complex disruption enables the neovascular buds originating from the choroid to pass through the disrupted layers into the sub-RPE or subretinal space. These immature vascular buds tend to thrive and subsequently leak and cause fluid leakage [6, 26, 27]. iCNV lesions often present with a new-onset metamorphopsia, vision loss and/or scotoma according to the location of CNV. However, the lesion sometimes may be asymptomatic, especially if located extrafoveally and can only be noticed during a routine fundus examination or imaging [6]. iCNV is often typically a type 2 CNV and can be peripapillar, extrafoveal, juxtafoveal or subfoveal. Rarely, iCNV presents as a type 1 CNV with or without a polypoidal component [6]. The active membrane is a grayish looking lesion and can have a hemorrhagic or exudative component. In the absence of significant exudation, sometimes the presence of a tiny intraretinal hemorrhage may indirectly point out to the membrane [4]. On the other hand, inactive or a healed membrane can be seen as a yellowish-white scar. As serous retinal detachment and intraretinal edema may also accompany an acute uveitis attack, the neovascular membrane may be easily overlooked and the clinical picture can be interpreted as the presentation of an uveitis attack [6]. Color fundus picture, fluorescein angiographic (FA), indocyanine green angiographic (ICGA) and optical coherence tomographic (OCT) images of a case with unilateral MFC together with a type 1 CNV and a polyp at the peripapillary area are shown in Figure 1.

**Figure 2:**
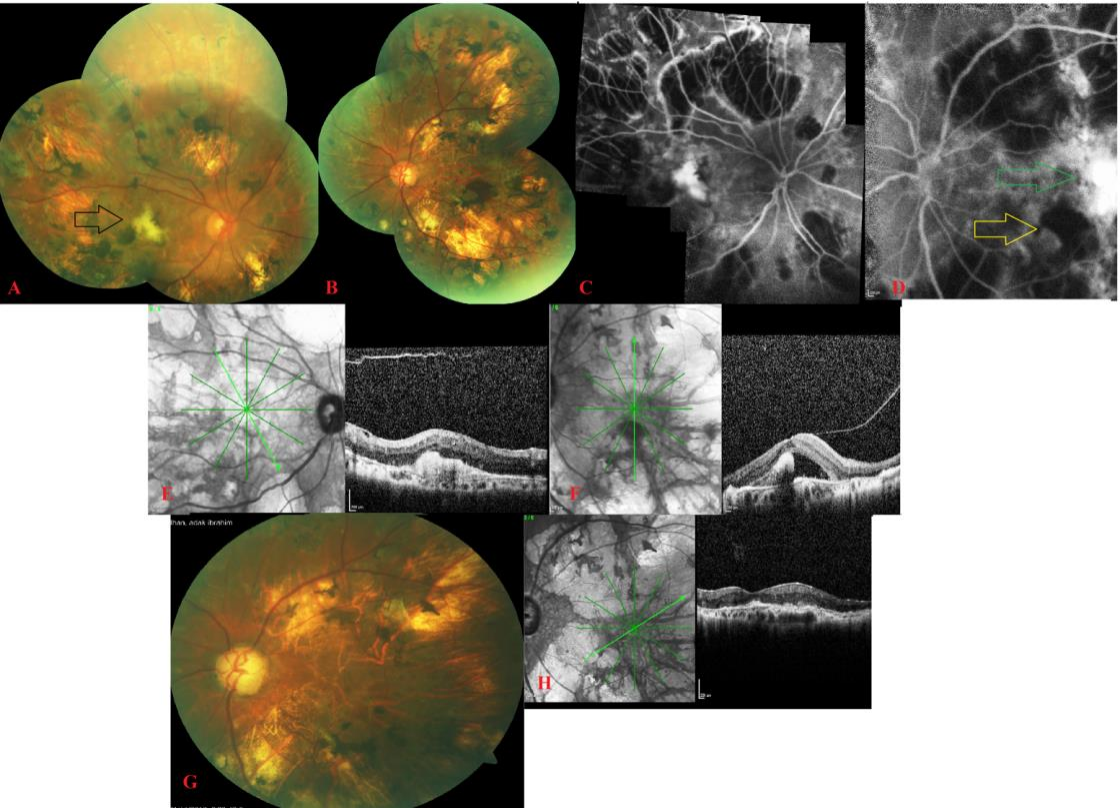
A 74-year-old male with bilateral old serpiginous like choroiditis scars and left active inflammatory choroidal neovascularization (iCNV) and a right disciform scar. Color fundus picture of the right (A) and left eyes (B) showing widespread healed choroiditis areas together with the scattered pigmentary changes. The right eye had a macular fibrotic scar (black arrow) and the left eye had a small grayish membrane (red arrow) with retinal hemorrhage. Late-phase fluorescein angiogram of the right (C) and left eyes (D) shows fluorescein blockage resulting from the widespread chorioretinal atrophic areas and pigment clumping. There was a hyperfluorescent foveal lesion with diffuse leakage (green arrow) corresponding to iCNV and a hypofluorescent area corresponding to hemorrhage (yellow arrow). OCT section shows a hyperreflective foveal scar in the right eye (E) and fluid accumulation with hyperreflective material in the subretinal space in the left eye (F). He was treated with four ranibizumab injections within one year. The iCNV healed with a scar formation and few residual persistent intraretinal cysts (G, H)

FA, ICGA and OCT are among the conventional imaging methods for determining the presence and location of CNV in eyes with uveitis [28]. Since iCNV is often a classic type of membrane, it can be visualized by FA [29-31]. However, ICGA may also be used if the lesion is an occult lesion as ICGA visualize the occult lesions much better than FA. As the iCNV also causes early iso- or hyper-fluorescence with late dye leakage [32], the presence of iCNV may be misrecognized as an active inflammatory lesion. On the other hand, OCT delineates the activity of iCNV by detecting the membrane itself and also accompanying intraretinal and/or subretinal fluid accumulation at the lesion area. Multimodal imaging of a 74-year-old male with a type 2 CNV associated with inactive bilateral serpiginous like choroiditis is demonstrated in Figure 2. 

Nowadays, OCT-Angiography (OCTA) is preferred for detection of CNV since vascular networks can be depicted many times by OCTA [33, 34] CNV is classified into three groups based on its visualization in OCTA; group A (well-circumscribed vascular complex), group B (moderately circumscribed vascular complex) and group C (poorly circumscribed vascular complex) [35].

Although various treatment modalities including vitreoretinal surgery, laser photocoagulation, corticosteroids (local and/or systemic), systemic immunosuppressive treatment and photodynamic therapy (PDT) have been used, VEGF inhibitors seem to become an important tool in the management of iCNV [2, 4, 6] and can be used alone or in conjunction with above-mentioned therapies. Also, there is an anectodal use of systemic sirolimus and intravitreal methotrexate injection [36, 37]. All therapeutic interventions essentially target neo-angiogenic and/or inflammatory mechanisms underlying iCNV development. Since even subclinical inflammation can provide a favorable environment for iCNV formation, suppression of any active inflammation is mandatory. Local and/or systemic steroids are used effectively alone or mostly in combination with immunosuppressive agents (cyclosporin, methotrexate and mycophenolate mofetil) and/or biologics (such as infliximab, adalimumab, etc.) to control the intraocular inflammation [4, 6, 38, 39]. Steroids can also be used either alone or combined with Photodynamic therapy (PDT) and/or anti-VEGF therapy. PDT has been used to treat subfoveal and/or juxtafoveal iCNV for various types of uveitis including MFC [40], PIC [41] and VKH [42] when there is no response with other treatment modalities prior to antiVEGF era. Subsequent subretinal fibrosis, atrophy and recurrence of iCNV restricted the use of PDT [28, 43]. Thermal laser photocoagulation can be an option for the treatment of extrafoveal iCNV in some selected cases[2]. This mini-review briefly summarizes the place of intravitreal anti-VEGF therapy in patients with non-infectious uveitis and iCNV.

## METHODS

An extensive literature search was performed in PubMed between 1979 and 2020 with the extracted keywords of ‘’inflammatory choroidal neovascularization'', ''intravitreal anti-VEGF therapy'', and ''non-infectious uveitis'') to retrieve suitable studies on intravitreal treatment in patients with iCNV associated with NIU. Studies regarding NIU-related iCNV and studies with sample size ≥ 5 eyes were evaluated.

**Intravitreal Anti-VEGF Therapy and Clinical Studies**

Bevacizumab (Avastin; Genentech Inc.), Ranibizumab (Lucentis; Genentech Inc., South San Francisco, California, The USA) and Aflibercept (Eylea; Regeneron Pharmaceutical Inc., Tarrytown, NY, The USA and Bayer Healthcare, Berlin, Germany) have been used successfully for treating many retinal diseases characterized by increased vascular permeability and/or neo-angiogenesis, such as exudative age-related macular degeneration (AMD), myopic CNV, macular edema secondary to retinal vein occlusion or diabetic retinopathy (DR), and proliferative DR [44]. Nowadays, anti-VEGF agents become an important therapeutic tool in patients with iCNV and are used alone or in combination with the above-mentioned treatment modalities. Table 1 summarizes the studies regarding the administration of anti-VEGF agents in the management of iCNV associated with NIPU [45-51].

Anti-VEGF therapy has gained popularity as promising outcomes have been reported in many studies conducted with bevacizumab, ranibizumab or aflibercept [3, 6, 45-52]. However, most of the studies were uncontrolled-retrospective studies involving small sample size or case series. Currently, there is still no well-accepted anti-VEGF treatment algorithm for iCNVs. In a very recent clinical trial conducted by Invernizzi et al. [3], the data of 24-month outcomes of anti-VEGF injections in 82 eyes with iCNV caused by infectious and non-infectious etiologies were retrospectively analyzed. Most patients had a non-infectious etiology [PIC/MFC (40 eyes), VKH (9 eyes), sarcoidosis (4 eyes) and intermediate uveitis (3 eyes)]. Patients were divided into 2 groups according to the treatment regimen: LOADING group (eyes treated with 3 anti-VEGF injections monthly then pro re nata (PRN) and PRN group (eyes treated with anti-VEGF injections PRN from the beginning). Patients received either anti-VEGF agents as monotherapy [bevacizumab (61 eyes), ranibizumab (3 eyes) and aflibercept (5 eyes)] or switch (any combination of anti-VEGF) therapy (13 eyes). The authors reported that visual acuity improved significantly in both groups during the study period compared to its baseline. However, there was a significantly higher mean number of injections in the LOADING group (4.5) than in the PRN group (2.5), but the CNV recurrence rate was the same. The authors emphasized success of anti-VEGF therapy in the treatment of iCNV and concluded that PRN regimen had similar efficacy with the LOADING regimen. Parallel results were reported in the MINERVA study investigating the efficacy and safety of ranibizumab in uncommon causes of CNVs and better visual outcomes were reported at month 2 with ranibizumab therapy compared to the sham group. Furthermore, improvement in visual acuity maintained until month 12. The MINERVA study group suggested that individualized PRN regimen was effective in achieving visual acuity gains, regardless of baseline visual acuity and underlying CNV etiology [53].

In addition to ideal protocol selection, switch therapy among anti-VEGF agents and recurrence rate are still not well-determined in cases with iCNV. The recurrence rate of iCNV has been reported between 0 and 50% in various studies [54-56]. Several studies have reported less mean or median number of injections (ranged; 1.5 to 3.5) in NIPU-related iCNV compared to patients with exudative AMD [45-51]. Also, several multicenter prospective studies on anti-VEGF therapy for exudative AMD have disclosed that AMD eyes often needed VEGF inhibitors for a very long time. Though the need for prolonged anti-VEGF injections seems to be less necessary in eyes with iCNV the ideal injection number remains still obscure in iCNV due to lack of long-term reports [6]. In a case series on anti-VEGF switch therapy, the authors found that sufficient response might not be obtained after switching to aflibercept from ranibizumab in patients with MFC and unresolving intraretinal fluid [57].

**Table 1:** The Summary of Studies on Inflammatory Choroidal Neovascularization Treatment with Intravitreal Vascular Endothelial Growth Factor Inhibitors in Non-infectious Uveitis

**Study design, sample size and follow-up **	**Type of uveitis** **(Number of eyes)**	**Anti-VEGF therapy ** **(Mean or median number of injections) **	**Neovascular membrane localization**		**Anatomical and/or functional outcomes, adverse event and recurrence**
**Barth et al. [** 45 **], (2018)**					
*Retrospective *16 patients *15 months (6-40)	PIC (16)	*Bevacizumab (n:13) Ranibizumab (n:2) Both (n:1) *3.5 (1-9)		8 subfoveal 7 juxtafoveal 1 extrafoveal	*BCVA improved in eight eyes, remained stable in four eyes and decreased in four eyes. BCVA improved from logMAR 0.36 to logMAR 0.32 *CFT decreased from 344 µm to 276 µm *Recurrence: 8 eyes (50%)
**Fine et al.[** 46 **], (2009)**					
*Retrospective *5 patients (6 eyes) * 41.5 weeks (25–69)	MFC (6)	*Bevacizumab (n: 5 eyes) Ranibizumab (n: 0) Both (n: 1 eye) *2.3 (1– 6).		NR	*Five of six eyes improved to 20/30 acuity or better at 6 months. *One eye decreased to 20/400 due to bevacizumab-associated RPE tear
**Doctor et al.[** 47 **], (2009)**					
*Retrospective *5 patients (6 eyes) *15.3 months	BSCR (1) VKH (1) MFCPU (1) SO (1) Idiopathic (2)	*Bevacizumab (n: 6 eyes) *2.7 (1-5)		5 subfoveal 1 juxtafoveal	*BCVA improved in 60% of patients at the last follow-up. *No adverse event
**Cornish et al.[** 48 **], (2011)**					
*Retrospective *9 patients (9 eyes) *14.9 months (11-25)	PIC (9 eyes)	*Bevacizumab (n: 6 eyes) Ranibizumab (n: 3 eyes) *2.9 (1-6)		NR	*BCVA improved from logMAR 0.38 to logMAR 0.12 *No adverse event
**Parodi et al.[** 49 **], (2014)**					
*Prospective *7 patients (7 eyes) *12 months	SC (7 eyes)	*Bevacizumab (n: 7 eyes) *1.5 (1-5)		1 subfoveal 6 juxtafoveal	*BVCA improved in 2 eyes, remained stable in 4 eyes and decreased in one eye. Mean BCVA: 0.50 logMAR at baseline and 0.48 logMAR at last visit. *Mean CFT decreased from 254 µm to 196 µm at the last visit. * No adverse event
**Menezo et al.[** 50 **], (2010)**					
*Retrospective *10 patients (10 eyes) *12.5 months (6-34)	PIC (10 eyes)	*Ranibizumab (n: 9 eyes) Bevacizumab (n: 0) Both (1 eye) *1.9 (1-5)		NR	*Nine eyes remained stable or improved their vision and one eye deteriorated. *Mean CFT decreased from 302 µm to 283 µm after anti-VEGF therapy * No adverse event
**Tran et al.[** 51 **], (2008)**					
*Retrospective *10 patients (10 eyes) *7.5 months	MFC (6 eyes) SO (2 eyes) SC (1 eye) VKH (1 eye)	*Bevacizumab (10 eyes) *2.5 (1-4)		8 subfoveal 2 juxtafoveal	*BVCA improved from logMAR 0.62 to logMAR 0.45 at last visit *CFT decreased from 326 µm to 267 µm at last visit * No adverse event

Abbreviations: VEGF: vascular endothelial growth factor; BVCA: best-corrected visual acuity; VKH: Vogt–Koyanagi–Harada; PIC: punctate inner choroidopathy; MFC: multifocal choroiditis; BSCR: birdshot chorioretinopathy; MFCPU: multifocal choroiditis and panuveitis; SO: sympathetic ophthalmia; SC: serpiginous choroiditis; NR: not reported; CFT: central foveal thickness; logMAR: logarithm of minimum angle of resolution; n: number.

**Figure 3:**
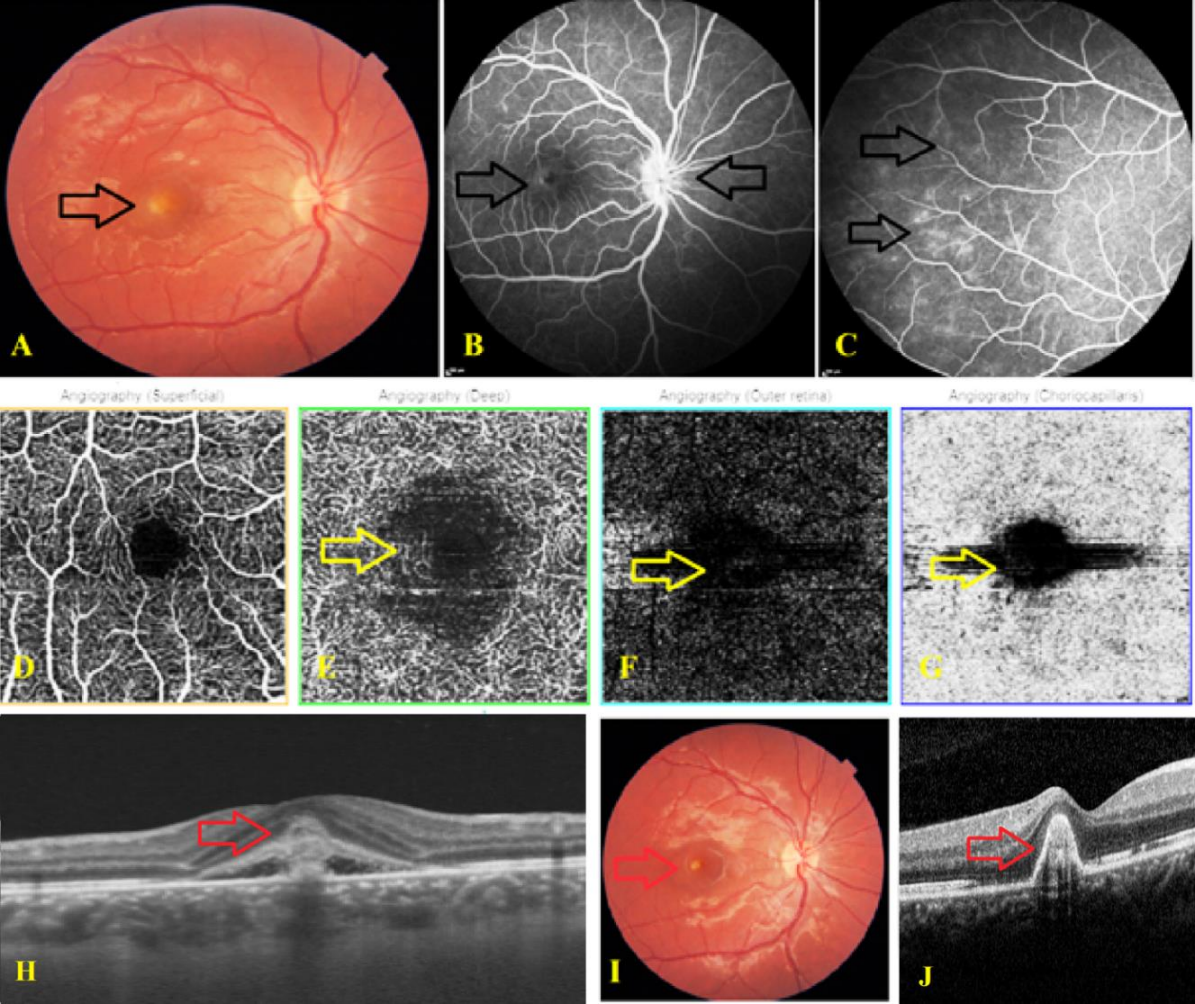
A 17-year-old girl with bilateral intermediate uveitis and right inflammatory choroidal neovascularization (iCNV). Color fundus picture (A) showing a subfoveal yellow-orange-colored lesion corresponding to pigment epithelial detachment (black arrow). Fundus angiogram (B, C) revealing ill-defined iCNV, disc staining and peripheral vascular leakage (black arrows). Optical coherence tomography angiography image of the superficial slab showing normal appearance (D), but deep (E), outer retina (F) and choriocapillaris (G) slabs revealing poorly circumscribed vascular complex with motion artifacts (yellow arrows). Optical coherence tomography (OCT) image (H) illustrating CNV and pigment epithelial detachment with pitchfork sign (red arrow). Four weeks after intravitreal injection of ranibizumab, color fundus photograph (I) and OCT image (J) showing partial regression of iCNV and resolution of subretinal fluid (red arrows)

Although iCNV is commonly associated with posterior uveitis, it can be rarely seen in intermediate uveitis (0.06%). The membrane is often located at the peripapillary area. The outcome of anti-VEGF treatment in this group is not well-known. However, the case series reported that iCNV in intermediate uveitis appears to respond well to anti-VEGF therapy [20, 58]. Unilateral iCNV and response to single intravitreal ranibizumab administration together with systemic steroid and azathioprine in a young girl with bilateral intermediate uveitis is illustrated in Figure 3.

Some clinicians may prefer a combination therapy of intravitreal VEGF inhibitors and oral or intravitreal steroids to avoid recurrence of iCNV and suppress the concomitant inflammation. In a retrospective study conducted by Wu et al.[59], twenty-four eyes of 22 patients with PIC and iCNV were treated with either intravitreal ranibizumab monotherapy (14 eyes) or combined oral corticosteroid and intravitreal ranibizumab therapy. Although visual improvement was achieved in both treatment groups, the authors reported that less development of new PIC lesion and CNV recurrence were observed in the combination group. Intravitreal steroids including triamcinolone acetonide (TA) and dexamethasone implant (IDI) have also been administered to obtain rapid remission avoiding possible systemic side effects of systemic steroid and/or immunosuppressive agents. Unfortunately, there are no randomized controlled trials about the efficacy of intravitreal steroids in NIU-related iCNVs. Pai et al. [60] reported that the combination of intravitreal TA and bevacizumab was an effective and reliable treatment alternative in VKH related recurrent iCNVs. Saatci et al. [16] administered a single simultaneous intravitreal dexamethasone implant (IDI) and ranibizumab injection in a case with active SC and iCNV and reported a favorable anatomic and functional outcome. Tsaousis et al. [61] obtained encouraging results and long-term stability with the use of IDI in patients with PIC-related iCNV.

## CONCLUSION

Various treatment options are implemented for the management of i-CNV associated with non-infectious type of uveitis. It is crucial to remember that underlying active or subclinical inflammation should be held under control as much as possible with steroids and/or immunosuppressive treatments or biologics where appropriate. However, locally targeted therapy is often given to obtain visually and anatomically satisfactory outcomes in many cases. Administration of anti-VEGF agents seems to be quite useful and characterized with a relatively low iCNV recurrence rate. However, randomized controlled trials with a larger sample size may be useful to search the optimal anti-VEGF treatment regimen and the superior anti-VEGF agent.

## DISCLOSURE

Ethical issues have been completely observed by the authors. All named authors meet the International Committee of Medical Journal Editors (ICMJE) criteria for authorship of this manuscript, take responsibility for the integrity of the work as a whole, and have given final approval for the version to be published. No conflict of interest has been presented. Funding/Support: None. The datasets analyzed during this study are available from the corresponding author on reasonable request.
